# Synthesis and Antibacterial Activities of Novel Imidazo[2,1-*b*]-1,3,4-thiadiazoles

**DOI:** 10.3390/molecules16075496

**Published:** 2011-06-28

**Authors:** Kamal F. M. Atta, Omaima O.M. Farahat, Alaa Z. A. Ahmed, Mohamed G. Marei

**Affiliations:** Chemistry Department, Faculty of Science, Alexandria University, Ibrahimia P.O. Box 426, Alexandria 21321, Egypt

**Keywords:** 1,3,4-thiadiazole, imidazo[2,1-*b*]thiadiazole, electrophile, thiosemicarbazide, phenacyl bromide, antimicrobial activity

## Abstract

2-Amino-5-(2-aryl-2*H*-1,2,3-triazol-4-yl)-1,3,4-thiadiazoles **2-4 **have been synthesized by the reaction of 2-aryl-2*H*-1,2,3-triazole-4-carboxylic acids **1** with thiosemicarbazide. Their reaction with phenacyl (*p*-substituted phenacyl) bromides led to formation of the respective 6-aryl-2-(2-aryl-2*H*-1,2,3-triazol-4-yl)imidazo[2,1-*b*]-1,3,4-thiadiazoles **5**. Reactivity of the latter fused ring towards reaction with different electrophilic reagents afforded the corresponding 5-substituted derivatives **6-8**. The structure of the above compounds was confirmed from their spectral characteristics. Some of these compounds were found to possess slight to moderate activity against the microorganisms *Staphylococcus aureus, Candida albicans, Pseudomonas aeruginosa, *and *Escherichia coli*.

## 1. Introduction

2-Amino-1,3,4-thiadiazole derivatives are well known as compounds of a wide range of anticancer activity [1,2,3,4] that have been prepared from their respective aldehydes [5] or carboxylic acids [6]. They appeared to be the most feasible route to fused imidazo[2,1-*b*]-1,3,4-thiadiazole rings [6,7,8,9,10,11,12,13,14] of interesting potential applications [15,16]. Some of these compounds show anticancer [17], antitubercular [18], antibacterial [19], antifungal [20], anticonvulsant analgesic [21], antisercetory [22] and antiapoptotic [23] properties. Also, certain compounds incorporating the imidazo[2,1-*b*]-1,3,4-thiadiazole moiety are used as anti-inflammatory [24], cardiotonic [25], diuretic [26] and herbicidal [27] agents and are used in the manufacture of dyes [28,29,30]. With all this in mind, the present paper focused on the synthesis of some new fused imidazo[2,1-*b*]-1,3,4-thiadiazole rings and testing their reactivity towards electrophilic substitution reactions and against *Staphylococcus aureus, Candida albicans, Pseudomonas aeruginosa *and *Escherichia coli*. 

## 2. Results and Discussion

**Scheme 1:**
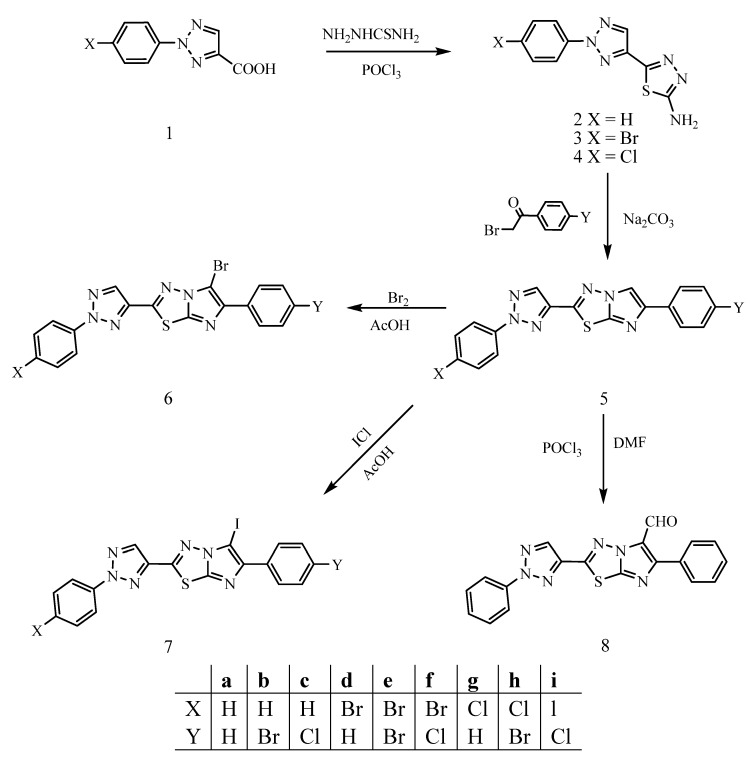
Synthesis and electrophilic substitution reactions of imidazo[2,1-b]-1,3,4-thiadiazoles.

2-Aryl-2*H*-1,2,3-triazole-4-carboxylic acids **1** [31] were chosen as starting materials for these studies. They were converted with thiosemicarbazide in the presence of POCl_3_ to the corresponding 2-amino-5-(2-aryl-2*H*-1,2,3-triazol-4-yl)-1,3,4-thiadiazoles **2-4** in good yields (Scheme 1). 

The structures of the above compounds were confirmed from their spectral data. Their IR spectra showed two medium intensity bands at range 3282–3485 and 3165–3300 cm^−1^ due to the asymmetric and symmetric NH stretching frequencies of the primary amino group [32,33]. 

**Table 1:** Infrared and ^1^H NMR spectral data of imidazo[2,1-*b*]-1,3,4-thiadiazoles (5-7).

Cpd. No.	IR (cm^−1^)					^1^H-NMR Chemical Shift (δ/ppm) *		
	C=N	C=N	C=N	C=C	C-S-C	ArH	Imidazole proton	Triazole proton
	(thiadiazole ring)	(imidazole ring)	(triazole ring)	(imidazole ring)	(thiadiazole ring)	(m)	(s, 1H)	(s, 1H)
**5a**	1638	1594	1534	1490	690	7.33–8.14 (10H)	8.07	8.33
**5b**	1635	1595	1532	1490	669	7.61–8.84 (9H)	7.50	8.46
**5c**	1634	1594	1536	1490	669			
**5d**	1629	1527	1540	1844	690			
**5e**	1631	1555	1528	1488	669			
**5f**	1626	1600	1529	1488	669	7.42–8.11 (8H)	8.06	8.50
**5g**	1651	1601	1538	1486	690			
**5h**	1649	1565	1533	1487	671			
**5i**	1652	1599	1533	1485	670			
**6a**	1640	1597	1545	1489	662	7.25–8.14 (10H)		8.41
**6b**	1641	1594	1522	1487	669	7.44–8.15 (9H)		8.42
**6c**	1637	1595	1524	1488	669	7.42–8.14 (9H)		8.41
**6d**	1632	1549	1526	1436	686	7.35–8.06 (9H)		8.42
**6e**	1632	1552	1522	1486	669	7.57–8.03 (8H)		8.42
**6f**	1628	1545	1521	1686	669	7.42–8.04 (8H)		8.42
**6g**	1645	1550	1510	1488	686	7.36–8.10 (9H)		8.42
**6h**	1642	1568	1519	1487	672	7.49–8.09 (8H)		8.41
**6i**	1641	1585	1522	1488	672	7.42–8.09 (8H)		8.41
**7a**	1634	1595	1532	1488	663	7.37–8.15 (10H)		8.43
**7b**	1640	1594	1527	1489	668	7.42–8.14 (9H)		8.41
**7c**	1636	1596	1530	1489	669	7.43–8.14 (9H)		8.41
**7d**	1642	1547	1522	1486	685			
**7e**	1633	1565	1535	1485	671	7.58–8.03 (8H)		8.41
**7f**	1633	1565	1521	1487	671	7.43–8.03 (8H)		8.42
**7g**	1639	1545	1509	1488	685	7.39–8.11 (9H)		8.42
**7h**	1640	1595	1555	1487	669	7.49–8.09 (8H)		8.41
**7i**	1639	1598	1565	1486	670	7.43–8.10 (8H)		8.41

*s = Singlet, m = multiplet.

**Figure 1:**
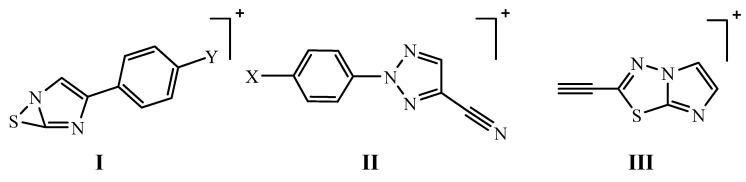
The most prominent fragments of the mass spectra of imidazo[2,1-*b*]-1,3,4-thiadiazoles.

Also, the ^1^H-NMR spectra revealed a singlet at δ 7.60–7.61 (2H) for exchangeable NH_2_ protons and a singlet at δ 8.51–8.52 for the H-5 triazole proton. The structures were further confirmed from mass spectral data (cf. Experimental). The reaction of 2-amino-5-(2-aryl-2*H*-1,2,3-triazol-4-yl)-1,3,4-thiadiazoles **2-4** with phenacyl(*p*-substituted phenacyl) bromides in boiling dry ethanol led to the formation of the new series of 6-aryl-2-(2-aryl-2*H*-1,2,3-triazol-4-yl)imidazo[2,1-*b*]-1,3,4-thiadiazoles **5a-i**. The reaction proceeds via the formation of the iminothiadiazole intermediate, followed by dehydrative cyclization to form the desired fused heterocycle [19]. The disappearance of the NH_2_ absorption bands in the IR spectra of **5** as well as the absence of the exchangeable singlets of this group in their ^1^H-NMR spectra, in addition to the presence of singlets at δ 8.33–8.50 for triazole protons, and a singlet at δ 7.50–8.07 for imidazole protons confirmed the structures (Table 1). It was reported that [27,28,29] the electrophilic substitution reactions of imidazo[2,1-*b*]-1,3,4-thiadiazole take place at position 5. Thus, in the present work, bromination of **5** with bromine as well as iodination with iodine monochloride gave the respective 5-bromo (**6a-i**) and 5-iodo derivatives **7a-i**. The ^1^H-NMR spectra of both derivatives revealed the disappearance of H-5 singlet of imidazole ring and presence of triazole proton as singlets at δ 8.41–8.43 (Table 1). The structure of the imidazothiadiazoles **5-7** was further confirmed by studying their mass spectral data, where the possible fragmentation pathways for all compounds gave rise to the bicyclic (I), 2-aryl-4-cyano-1,2,3-triazole (II). In addition to 2-acetylenic imidazo-thiadiazoles (III) (Figure 1). The relative intensity of the molecular ion peaks and the most prominent peaks are listed in (Table 2). 

**Table 2:** Relative intensity of the molecular ion peaks and the most prominent peaks in the mass spectra of imidazo[2,1-*b*]-1,3,4-thiadiazoles (5-7).

Cpd. No.	M	I	II	III
**5a**	33	2	11	8
**5b**	100,78	10	70	5
**5g**	21	5	36	4
**5i**	29	24	40	4
**6a**	69	100	34	18
**6d**	7	57	100	12
**6f**	8	40	8	7
**6g**	22	100	29	19
**6i**	5	35	34	1
**7a**	100	73	22	18
**7e**	39	16,15	44,46	2
**7g**	46	100	18	57
**7i**	14	36	18	1

Formylation of **5a** with dimethylformamide in the presence of phosphorus oxychloride (Vilsmeier-Haack reagent) gave the 5-formyl derivative **8** in excellent yield. Its IR spectrum showed a strong absorption band at 1676 cm^−1^ characteristic of the entered carbonyl group and two weak absorption bands at 2921 and 2885 cm^−1^ of formyl hydrogen. Also, the ^1^H-NMR spectrum revealed the absence of the H-5 singlet of the imidazole ring and exhibited two singlets at δ 8.53 and 10.10 for the triazole and formyl protons respectively.

### Biological screening: antimicrobial activity tests

The antibacterial activities of compounds **2-8** were tested against the micro-organisms *Staphylococcus aureus (ATCC6538p), Candida albicans (ATCC2091), Pseudomonas aeruginosa (ATCC9027) *and *Escherichia coli *(ATCC8739). After incubation, the diameters of inhibition zones around the wells were measured, to the nearest mm, in three different directions using a ruler and the average diameter was recorded and compared to that of the control. From the data presented in Table 3 it is clear that compounds **5d** and **6h** were moderately active against *Escherischia coli, *while **7b** was also moderately active but against *Pseudomonas aeruginosa* microorganisms. Generally, other derivatives were slightly too moderately active against *Candida albicans*, *Pseudomonas aeruginosa* and *Escherischia coli*. Finally, compounds **2–8** were found to be inactive against *Staphylococcus aureus*.

**Table 3:** Result of antimicrobial activity tests (agar diffusion method) of compounds (2–8).

Test microorganisms	*S*	*C*	*Ps*	*E*
Test compounds	Average inhibition zone diameter in mms			
**Imepenam**	30	-	30	26
**Ampicillin**	30	-	-	-
**Clotrimazole**	-	40	-	-
**2**	-	18	22	17
**3**	-	18	22	16
**4**	-	18	22	16
**5a**	-	18	22	17
**5b**	-	18	22	18
**5c**	-	18	22	16
**5d**	-	18	22	20
**5e**	-	18	22	16
**5f**	-	18	22	18
**5g**	-	18	22	16
**5h**	-	19	22	18
**5i**	-	18	23	16
**6a**	-	19	22	17
**6b**	-	18	23	16
**6c**	-	18	22	17
**6d**	-	18	23	17
**6e**	-	18	22	16
**6f**	-	18	22	17
**6g**	-	18	22	17
**6h**	-	18	22	20
**6i**	-	18	22	16
**7a**	-	18	22	19
**7b**	-	18	26	19
**7c**	-	18	22	16
**7d**	-	18	22	16
**7e**	-	19	22	17
**7f**	-	19	22	17
**7g**	-	19	22	16
**7h**	-	18	22	17
**7i**	-	18	22	16
**8**	-	18	22	19
**DMF **	-	18	22	16

*S:* *Staphylococcus aureus; C: Candida albicans; Ps: Pseudomonas aeruginosa; E: Escherichia coli.*

## 3. Experimental

### 3.1. General

Melting points were determined on a Kofler Block and are uncorrected. TLC was done on Merck Kiesel gel 60-f 254 precoated plastic plates. Infrared spectra (IR) were measured with a Fourier Transform Infrared 8400 spectrophotometer (BRUKER TENSOR 37) using potassium bromide pellets. The ^1^H-NMR spectra were recorded on a JEOL JNM ECA 500 MHZ with tetramethylsilane as internal standard. Mass spectra (MS) were recorded at 70 ev using GCMS-QP 1000EX mass spectrometer. Microanalyses were performed by the Microanalytical Unit, Cairo University, Cairo. 2-Aryl-2H-1,2,3-triazole-4-carboxylic acids **1** were prepared from the respective D-glucose arylosotriazoles as described earlier [31].

#### 3.2.1. 2-Amino-5-(2-aryl-2H-1,2,3-triazol-4-yl)-1,3,4-thiadiazoles **(2-4)**

2-Aryl-2*H*-1,2,3-triazole-4carboxylic acids (**1**, 0.1 mol) and thiosemicarbazide (9.1 g, 0.1 mol) were refluxed gently in the presence of phosphorus oxychloride (30 mL) for 45 min. The reaction mixture was cooled and quenched with cold water (90 mL). The resulting solution was refluxed for an additional 6 hours. The separated solid was filtered and suspended in water and basified with potassium hydroxide. The solid was filtered, washed with water, dried and crystallized from a suitable solvent.

*2-Amino-5-(2-phenyl-2H-1,2,3-triazol-4-yl)-1,3,4-thiadiazole* (**2**). Yield (75%); m.p. 251–252 °C (crystallization from ethanol); IR (cm^−1^): 3282, 3165 (NH_2_), 1637 (thiadiazole ring C=N), 1555 (triazole ring C=N) and 690 (thiadiazole ring C-S-C); ^1^H-NMR (DMSO-d_6_, δ, ppm): 7.42 (t, 1H, aromatic-H), 7.55 (t, 2H, aromatic-H), 7.60 (s, 2H, exchangeable NH_2_), 7.98 (d, 2H, aromatic-H) and 8.51 (s, 1H, triazole-H); MS, *m/z* (%): 244 (100, M+), 188 (23, M+-C_2_H_2_N_3_), 161 (3, M+-C_2_H_3_N_4_), 145 (2, M+-C_2_HN_3_S), 105 (3, M+-C_4_H_3_N_4_S) and 91 (21, M+-C_2_H_3_N_5_S); Anal. Calc. for C_10_H_8_N_6_S (244.28): C, 49.17; H, 3.30; N, 34.40%, found: C, 49.00; H, 3.45; N, 34.25%.

*2-Amino-5-[2-(4-bromophenyl)-2H-1,2,3-triazol-4-yl]-1,3,4-thiadiazole* (**3**). Yield (77%); m.p. 259–260 °C (crystallization from dioxane); IR (cm^−1^): 3485, 3300 (NH_2_), 1639 (thiadiazole ring C=N), 1597 (triazole ring C=N) and 660 (thiadiazole ring C-S-C); ^1^H-NMR (DMSO-d_6_δ, ppm): 7.55 (d, 2H, aromatic-H), 7.61 (s,2H, exchangeable NH_2_), 7.98 (d, 2H, aromatic-H) and 8.52 (s, 1H, triazole-H); MS, *m/z* (%): 324 (8, M+ +2), 322 (5, M+), 266 (3, M+-CH_2_N_3_), 239 (1, M+-C_2_H_3_N_4_), 244 (100, M+-Br+H), 188 (54, M+-CHBrN_3_), 184 (3, M+-C_4_H_2_N_4_S), 169 (4, M+-C_4_H_3_N_5_S),161 (1, M+-C_2_H_2_BrN_4_), 145 (1, M+-C_2_BrN_3_S), 133 (2, M+-C_3_BrN_3_S), 105 (4, M+-C_4_H_2_BrN_4_S) and 91 (9, M+-C_2_HBrN_5_S); Anal. Calc. for C_10_H_7_BrN_6_S (323.17): C, 37.17; H, 2.18; N, 26.00%, found. C, 37.00; H, 2.60; N, 25.70%.

*2-Amino-5-[2-(4-chlorophenyl)-2H-1,2,3-triazol-4-yl]-1,3,4-thiadiazole* (**4**). Yield (65%); m.p. 269–270 °C (crystallization from dioxane); IR (cm^−1^): 3465, 3290 (NH_2_), 1626 (thiadiazole C=N), 1532 (triazole C=N) and 694 (thiadiazole C-S-C); Anal. Calc. for C_10_H_7_ClN_6_S (278.72): C, 43.09; H, 2.53; N, 30.15%, found. C, 43.12; H, 2.51; N, 30.17%. 

#### 3.2.2. 6-Aryl-2-(2-aryl-2H-1,2,3-triazol-4-yl)imidazo[2,1-b]-1,3,4-thiadiazoles **5a-i**

A mixture of equimolar quantities of thiadiazoles **2-4** (0.01 mol) and phenacyl (*p*-substituted phenacyl) bromides (0.01 mol) was refluxed in dry ethanol (50 mL) for 24 hours. The excess of solvent was distilled off and the solid hydrobromide salt that separated out was collected by filtration, suspended in water and neutralized by sodium carbonate to get free base **5a-i**. The product was filtered, washed with water, dried and crystallized from carbon tetrachloride. Their IR, ^1^H-NMR spectra and analytical data are listed in Table 1 and Table 4.

#### 3.2.3. 6-Aryl-2-(2-aryl-2H-1,2,3-triazol-4-yl)-5-haloimidazo[2,1-b]-1,3,4-thiadiazoles **6a-i** and **7a-i**

A solution of bromine (iodine monochloride) (0.01 mol) in acetic acid (10 mL) was gradually added to a suspension of **5a-i** (0.01 mol) in acetic acid (10 mL) with stirring for one to eight days at room temperature. The reaction mixture was poured onto ice cold water. The separated solid was collected, washed with water, dried and crystallized from either chloroform or benzene in methanol mixture. Their IR, ^1^H-NMR spectra and analytical data are listed in Table 1 and Table 4.

**Table 4:** Analytical data of imidazo[2,1-*b*]-1,3,4-thiadiazoles (**5-7**).

Cpd. No.	Mp (°C)	Yield (%)	Formula	Calcd. %			Found %		
				C	H	N	C	H	N
**5a**	225-226	60	C_18_H_12_N_6_S	62.77	3.51	24.40	62.50	3.42	24.43
**5b**	253-254	62	C_18_H_11_BrN_6_S	51.07	2.62	19.85	51.00	2.32	19.92
**5c**	257-258	61	C_18_H_11_ClN_6_S	57.07	2.93	22.18	57.11	2.86	22.20
**5d**	285-286	61	C_18_H_11_BrN_6_S	51.07	2.62	19.85	51.15	2.40	20.06
**5e**	281-282	60	C_18_H_10_ Br_2_N_6_S	43.05	2.01	16.73	43.07	2.00	16.81
**5f**	276-277	66	C_18_H_10_BrClN_6_S	47.23	2.20	18.36	47.31	2.12	18.02
**5g**	279-280	58	C_18_H_11_ClN_6_S	57.07	2.93	22.18	57.10	2.92	22.21
**5h**	267-268	55	C_18_H_10_BrClN_6_S	47.23	2.20	18.36	47.19	2.26	18.41
**5i**	271-272	58	C_18_H_10_ Cl_2_N_6_S	52.31	2.44	20.33	52.40	2.40	20.21
**6a**	221-222	80	C_18_H_11_BrN_6_S	51.07	2.62	19.85	51.12	2.42	20.03
**6b**	261-262	80	C_18_H_10_ Br_2_N_6_S	43.05	2.01	16.73	43.12	2.00	16.92
**6c**	255-256	80	C_18_H_10_BrClN_6_S	47.23	2.20	18.36	47.21	2.15	18.38
**6d**	265-266	82	C_18_H_10_ Br_2_N_6_S	43.05	2.01	16.73	43.05	2.01	16.73
**6e**	246-247	76	C_18_H_9_ Br_3_N_6_S	37.21	1.56	14.46	37.22	1.81	14.51
**6f**	248-249	80	C_18_H_9_Br_2_ClN_6_S	40.29	1.69	15.66	40.01	1.81	15.62
**6g**	257-258	80	C_18_H_10_BrClN_6_S	47.23	2.20	18.36	47.12	2.25	18.39
**6h**	259-260	80	C_18_H_9_Br_2_ClN_6_S	0.29	1.69	15.66	40.42	1.72	15.52
**6i**	237-238	80	C_18_H_9_ BrCl_2_N_6_S	43.93	1.84	17.08	43.90	1.87	16.90
**7a**	270-271	85	C_18_H_11_IN_6_S	45.97	2.36	17.87	45.82	2.39	17.85
**7b**	251-252	83	C_18_H_10_BrIN_6_S	39.37	1.84	15.30	39.40	1.90	15.21
**7c**	251-252	82	C_18_H_10_ClIN_6_S	42.83	2.00	16.65	42.90	2.03	16.82
**7d**	273-274	85	C_18_H_10_BrIN_6_S	39.37	1.84	15.30	39.21	1.61	15.42
**7e**	251-252	80	C_18_H_9_Br_2_IN_6_S	34.42	1.44	13.38	34.50	1.49	13.42
**7f**	243-244	85	C_18_H_9_BrClIN_6_S	37.04	1.55	14.40	37.24	1.52	14.43
**7g**	285-286	82	C_18_H_10_ClIN_6_S	42.83	2.00	16.65	42.72	1.95	16.62
**7h**	255-256	84	C_18_H_9_BrClIN_6_S	37.04	1.55	14.40	36.92	1.61	14.41
**7i**	253-254	80	C_18_H_9_Cl_2_IN_6_S	40.10	1.68	15.59	40.20	1.72	15.51

#### 3.2.4. 5-Formyl-6-phenyl-2-(2-phenyl-2H-1,2,3-triazol-4-yl)imidazo[2,1-*b*]-1,3,4-thiadiazole **(8)**

The Vilsmeir-Haack reagent was prepared by adding phosphorus oxychloride (3 mL) to dimethyl-formamide (20 mL) at 0 °C with stirring. Then, compound **5a **(3.44 g, 0.01 mol) was added to the reagent and stirred at 0 °C for 30 min. The mixture was further stirred at room temperature for 2 hours and then at 60 °C for an additional 2 hours. The reaction mixture was then poured onto sodium carbonate solution and stirred at 90 °C for 2 hours. After cooling, the mixture was diluted with water and extracted with chloroform. The chloroform layer was washed with water, dried over anhydrous sodium sulfate and the residue obtained after removal of the solvent was crystallized from chloroform/ methanol. Yield (80%); m.p. 184–185 °C; IR (cm^−1^): 2921, 2885 (formyl hydrogen), 1676 (CO), 1629 (thiadiazole C=N), 1589 (imidazole C=N), 1492 (triazole C=N), 1444 (imidazole C=C) and 664 (thiadiazole C-S-C); ^1^H-NMR (CDCl_3,_δ, ppm): 7.44 (t, 1H, aromatic-H), 7.54 (m, 5H, aromatic-H), 7.89 (d, 2H, aromatic-H), 8.15 (d, 2H, aromatic-H), 8.53 (s, 1H, triazole-H) and 10.1 (s, 1H, formyl-H); Anal. Calc. for C_19_H_12_N_6_OS (372.40): C, 61.28; H, 3.25; N, 22.57%, found. C, 61.30; H, 3.30; N, 22.57%.

### 3.3. Antibacterial Activity Tests

The antibacterial activity tests were performed according to agar diffusion method [34] using ampicillin, imepenam and clotrimazole as the reference compounds. The Sterile cotton swabs were separately dipped into each of the adjusted organism cultures and excess inoculum was removed by pressing and rotating the swab firmly several times against the wall of the tube above the level of the liquid. The swab was streaked all over the surface of the nutrient agar in three dimensions at an angle of 60° to obtain an even distribution of the inoculum. The plates were then left to dry at room temperature for few minutes. A sterile cork porer (8 mm in diameter) is used to make wells in the solid nutrient agar plates, so that the distance between the edges of each two wells is not less than 24 mm. Fill each well with 75 μL of the test compound and another well with same volume of DMF as a vehicle control. Allow a period of free diffusion for 2 h, then incubated at 37 °C for 18–24 h. 

## 4. Conclusions

In conclusion, formation of diverse 2-amino-5-substituted-1,3,4-thiadiazoles using carbohydrates as precursors was successfully achieved, followed by their conversion into the novel target imidazo[2,1-*b*]-1,3,4-thiadiazole fused heterocyles. The antibacterial activities of the synthesized compounds were also examined.
